# The Influence of Different Plasma Cell Discharges on the Performance Quality of Surgical Gown Samples

**DOI:** 10.3390/ma14154329

**Published:** 2021-08-03

**Authors:** Atif H. Asghar, Ahmed Rida Galaly

**Affiliations:** 1 Department of Environmental and Health Research, The Custodian of the Two Holy Mosques Institute for Hajj and Umrah Research, Umm Al-Qura University, Makkah 24381, Saudi Arabia; ahasghar@uqu.edu.sa; 2 Department of Physics, Faculty of Science, Beni-Suef University, Beni-Suef 62521, Egypt; 3 Department of Engineering Science, Faculty of Community, Umm Al-Qura University, Makkah 24381, Saudi Arabia

**Keywords:** DC glow discharge, different cathode configurations, cathode fall thickness, floating potential, surgical gown sample, wettability

## Abstract

An experimental study was performed on a low-density plasma discharge using two different configurations of the plasma cell cathode, namely, the one mesh system electrodes (OMSE) and the one mesh and three system electrodes (OMTSE), to determine the electrical characteristics of the plasma such as current–voltage characteristics, breakdown voltage (V_B_), Paschen curves, current density (J), cathode fall thickness (d_c_), and electron density of the treated sample. The influence of the electrical characteristics of the plasma fluid in the cathode fall region for different cathode configuration cells (OMSE and OMTSE) on the performance quality of a surgical gown was studied to determine surface modification, treatment efficiency, exposure time, wettability property, and mechanical properties. Over a very short exposure time, the treatment efficiency for the surgical gown surface of plasma over the mesh cathode at a distance equivalent to the cathode fall distance d_c_ values of the OMTSE and for OMSE reached a maximum. The wettability property decreased from 90 to 40% for OMTSE over a 180 s exposure time and decreased from 90 to 10% for OMSE over a 160 s exposure time. The mechanisms of each stage of surgical gown treatment by plasma are described. In this study, the mechanical properties of the untreated and treated surgical gown samples such as the tensile strength and elongation percentage, ultimate tensile strength, yield strength, strain hardening, resilience, toughness, and fracture (breaking) point were studied. Plasma had a more positive effect on the mechanical properties of the OMSE reactor than those of the OMTSE reactor.

## 1. Introduction

For 50 years, the direct current (DC) glow discharge has actively contributed to the fundamental phenomena [1,2] of practical plasma processes that modify material properties, such as plasma–surface modification, plasma polymerization, sterilization, and industrial applications, more so than radio frequency (RF) power sources [3,4].

DC cold plasma technologies using low-density weakly ionized argon plasma have been widely used in chemical, physical, and biological applications because of their surface modification effect. Controlling the current density by different techniques in glow discharge plasma is an important factor in tool heating, sputtering, etching, coating, disinfection processes, and ionization [5].

The basic techniques for the detection of small amounts of Ar plasma in industry, such as coating or etching, have been developed and improved [6].

The influence of configurations; electrode design parameters (cathode geometries, mesh cathode, hollow cathode, magnetized cathode, cavity cathode, etc.); and parameters of the plasma reactor such as the ion velocity, plasma density distribution, plasma kinetics, performance near the emission boundary, gas type, frequency, and flow rates have been investigated in studies related to surgical gown quality. Medical applications such as the etching process, coating process, and inactivation of microbial processes have also been investigated [7,8,9].

Discharges in a low-density weakly ionized argon plasma were briefly discussed and investigated in theoretical and experimental studies of radial electron temperature profiles [10] and by determination of the cathode fall thickness in the magnetized and unmagnetized DC plasma [11,12].

One important safety requirement for the healthcare surgical team is the surgical gown. Many articles have dealt with the performance of surgical gowns regarding their resistance to liquid penetration, water repellency, prevention of bacterial infections, and pathogen resistance of the fabric, in order to improve the mechanical properties of the samples [13].

Plasma treatment is widely used to treat inorganic and organic surfaces in the deposition of thin films and processing of materials. The surface modification of polymer films by plasma is the most effective method of uniform and controlled treatment. The surface energy of the films is controlled to enhance the wettability and adhesion of coatings by plasma treatment under different processing conditions [14].

The plasma treatment of textiles is a more efficient technology than traditional industry methods, which produce large amounts of liquid wastes that contain organic and inorganic compounds. Treatment of textiles with plasma, which is considered to be an environmentally acceptable physical agent, includes applications such as the treatment of surgical gowns to enhance the adhesion of reduced graphene oxide for electro-conductive properties [15], plasma sputtering of copper on polyester/cotton blended fabrics for the creation of multifunctional properties [16], structural and characteristic changes of water hyacinth fiber from the combined effect of plasma and nano-technology [17], surface and moisture characterization of jute using a DC glow discharge argon plasma [18], and the single-step approach of fabricating superhydrophobic PET fabric using low pressure plasma for oil–water separation [19].

Plasma technology is being developed for many reasons in the textile industry, such as antimicrobial properties, self-cleaning, flame resistance, resistance to ultraviolet degradation, antistatic properties, water repellency, and dimensional stability of material [20]. Exposure to DC plasma can cause chemical and physical changes in the surgical gown surface or near-surface layers. Reactive species generated in the DC glow discharge [21] produce more reactive surfaces and affect wettability, stress, and strain properties. Advances in modern textile technology processes are attributed to the increasing demands of the environment.

The objective of this study was to construct two different plasma reactors to analyze, study, and discuss the plasma treatment of surgical gowns, in order to investigate the creation of multifunctional properties of surface modification. Plasma treatment offers different industrial applications and increases the performance quality, the treatment efficiency, and the mechanical properties of the surgical gown. It also decreases the wettability property and the exposure treatment time, as well as eliminating the spread of microbes.

In the present work, the electrical characteristics of the low-pressure glow discharge of the DC (cold cathode) sputtering unit for different configurations of the plasma reactor cathode were investigated to determine the experimental current density of the cathode fall region. The optimum distance of the cathode fall region (d_c_) was studied through a comparison between the theoretical and experimental results of the current density. For the different systems (OMSE and OMTSE), an experimental study was conducted on characteristics such as electron density, electron temperatures, floating potential, treatment efficiency, exposure time, wettability property, and mechanical properties of DC plasma for surgical gown treatment. At optimum distance of the cathode fall region (d_c_), a comparison was made between surgical gown samples placed at different distances with respect to the mesh cathode and subjected to different exposure times to investigate the wettability of the surgical gown surface.

## 2. Experimental Set-Up

### 2.1. System Preparations

**Figure 1:**
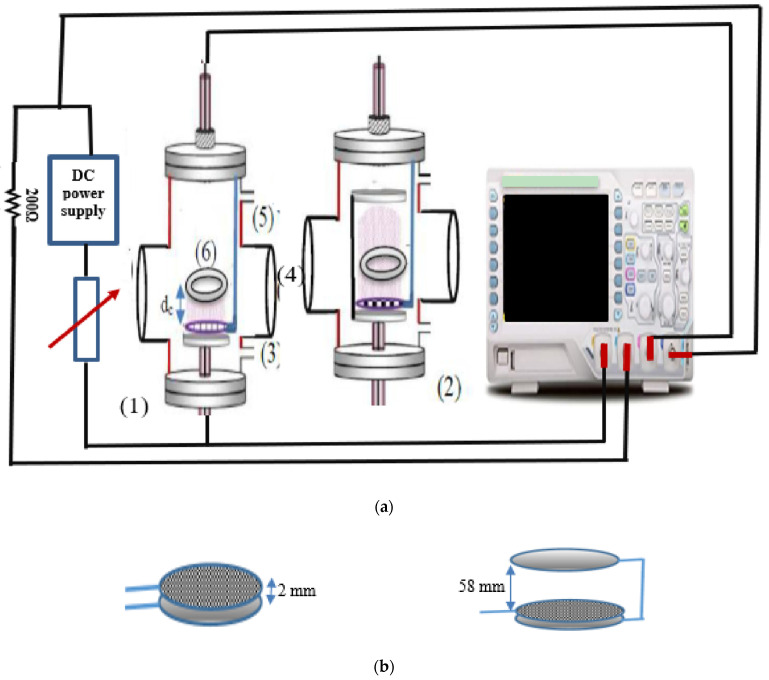
(**a**) Schematic diagram of the experimental set-up of the evacuated chamber containing (**1**) the OMSE reactor, (**2**) the OMTSE reactor, (**3**) gas inlet, (**4**) window, (**5**) rotary pump, and (**6**) sample holder over the cathode fall region dc. (**b**) System 1, OMSE reactor (**left**), and System 2, OMTSE reactor (**right**).

Figure 1a shows a stainless-steel chamber with glass windows that was evacuated to 7 mTorr with a two-stage rotary pump. High purity Ar working gas was fed into the chamber through a needle valve. A stationary DC glow discharge was generated between two electrodes of metallic disks for the different designs and for different low Ar pressures using a 1200-volt DC power supply. The applied voltage and discharge currents were measured with a Tektronix digital oscilloscope. The discharge current ranged from 4 to 90 mA, the gas pressure ranged from 0.5 to 5 mTorr, the discharge voltage ranged from 100 to 1200 V, and the current density ranged from 2 to 15 mA/cm^2^.

Figure 1 shows the schematic diagram of the experimental set-up of the electrical circuit established to create a glow discharge inside the evacuated chamber between two different electrode configurations of the plasma cell, which were used earlier by the author [22,23] as follows:

System 1, called the OMSE reactor, consisted of two parallel circular electrodes in the axial position: one aluminum cathode mesh electrode and a copper electrode working as anode placed below the cathode at a gap distance of 2 mm, enough to prevent a plasma forming between them. System 2, called the OMTSE reactor, consisted of three parallel circular electrodes in the axial position; two copper anode plates (separated by 60 mm); and an Al mesh electrode working as a cathode placed between the two copper anodes, 2 mm above the first anode and 58 mm below the second anode.

The grounded holders for the surgical gown samples, mesh cathode, anode, and the two systems (OMSE and OMTSE) were isolated from the stainless-steel outer chamber by *polytetrafluoroethylene* (*PTFE*)-insulated material to prevent the build-up of charged sheaths on their surfaces and confine the plasma over the cathode mesh, as well as to strengthen the plasma outside the cathode mesh.

### 2.2. Textile Preparations

**Figure 2:**
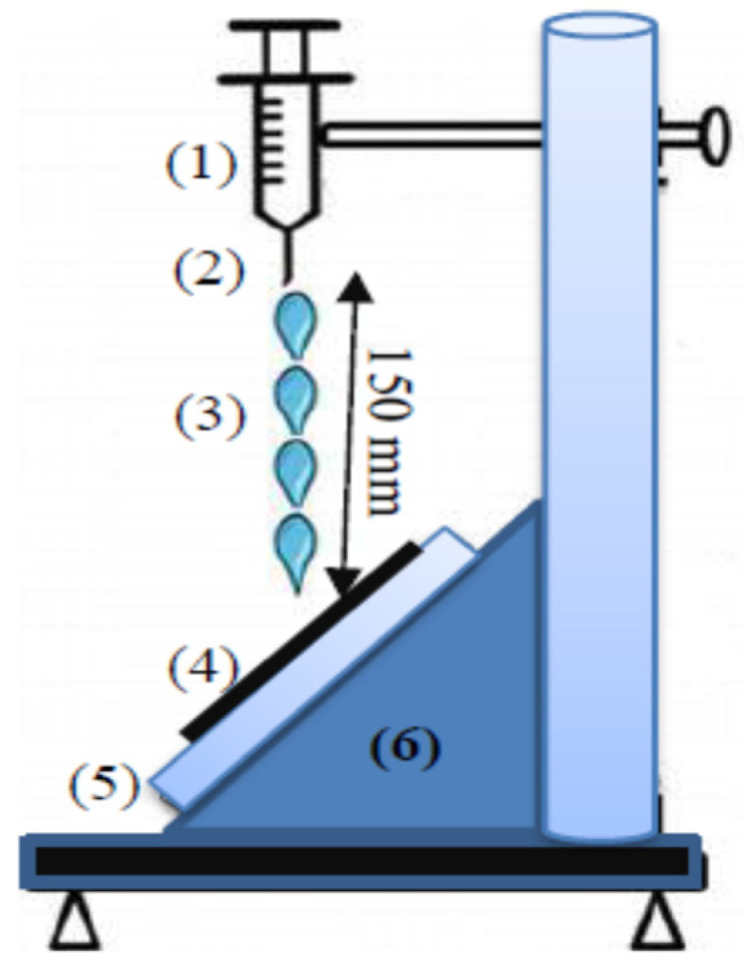
Schematic diagram for the water repellency spray tester containing (**1**) syringe, (**2**) jet nozzle, (**3**) water drop, (**4**) fabric sample, (**5**) support inclined at 45°, and (**6**) holder.

Parameters of performance and quality were measured for the surgical gowns treated with a DC glow discharge at low gas pressure (1 mTorr), as well as for different cathode configurations, cathode fall thicknesses, and treatment exposure times (t). Figure 2 shows “*the water repellency test*” for the wettability measurements of the cotton textile before and after plasma treatment. This test measured the wettability percentage and the state of water repellency (waterproof) of the textiles wetted with a syringe filled with 250 mL of water at room temperature, through a jet nozzle of 6.3 mm diameter, separated by an axial distance of 150 mm from the fabric sample, which was mounted on an inclined holder sloping at an angle of 45° for 25 s. The percentage of free water clinging to the fabric sample was then measured [24,25].

The tensile and the elongation behaviors were tested for the surgical gown samples, untreated and treated with the two different plasma reactors (OMSE and OMTSE), *using Zweigle Model Z010 according to ASTM D412-98a under the standard atmospheric conditions and at a tension speed of 100 mm/min*, wherein the measurements were carried out three times, and the results represented the mean values. The mechanical properties of the untreated and treated samples were tested with a uniform DC glow discharge, indicated by the stress  σ (KPa) as a function of the strain  ε  (percent), where  σ=E ε, with *E* representing Young’s modulus (stiffness) values.

The present work focused on the effect of different cathode configurations on two types of plasma reactors (OMSE and OMTSE). At a distance equivalent to d_c_, different surgical gown samples were placed on a holder apart from the mesh cathode, where the effect of current density on the wettability rate was measured for different exposure times and cathode configurations of the plasma cell to investigate the surface treatment using a DC glow discharge.

## 3. Results and Discussion

### 3.1. The Characteristics of Different Cathode Configurationtables

The performance of the two reactors (OMSE and OMTSE) depended on the configuration of the cathode mesh in the plasma cell using the DC glow discharge. The uniform argon plasma discharges in the OMSE and OMTSE reactors were compared by a study of electrical characteristics such as current–voltage, breakdown voltage (V_B_), Paschen curves, current density (J), and cathode fall thickness (d_c_) as follows.

#### 3.1.1. I–V Characteristics

**Figure 3:**
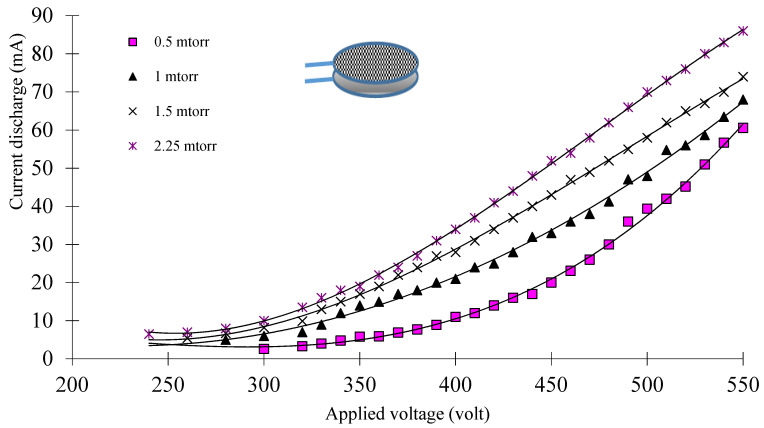
Characteristic I–V curve of argon gas discharge at different pressures and applied voltages, using the OMSE configuration system.

**Figure 4:**
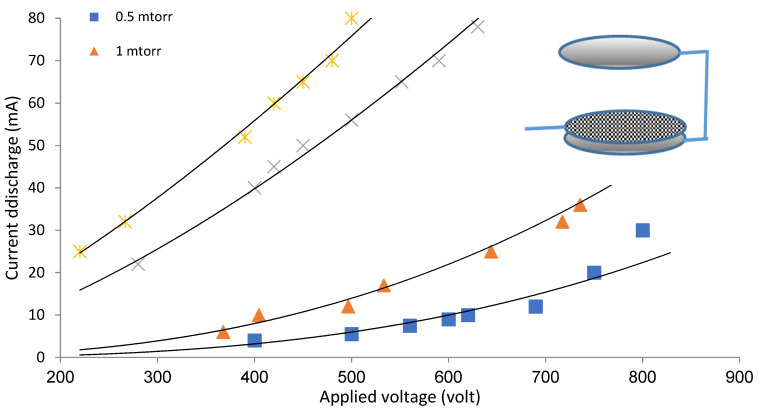
Characteristic I–V curve of argon gas discharge at different pressures and applied voltages, using the OMTSE configuration system.

Figure 3 and Figure 4 show the I–V characteristic curves of the low-density plasma using a weakly ionized argon gas discharge at different pressures and applied voltages for the two different configurations systems (OMSE and OMTSE), respectively.

By increasing the gas pressure from 0.5 to 2.25 mTorr, the discharge current increased, and the characteristic curves confirmed that the electrical discharge was mainly in the abnormal glow discharge region for both reactors (OMSE and OMTSE). The breakdown voltage of the discharge decreased when increasing the gas pressure at a constant discharge current. This may be related to the fact that when the gas pressure increased, the mean free path $\lambda_{e-n}$ decreased [26]; hence, more excitation and ionization processes occurred and, consequently, the starting potential decreased, where $\lambda_{e-n}$ is inversely proportional to the gas pressure, as in Equation (1):

(1) $$\lambda_{e-n}=\frac{1}{3.55\times 10^{16}\;P\;Q_{i}}$$

where $\lambda_{e-n}$ is the mean free path, *P* is the gas pressure in Torr, and $Q_{i}$ is the ionization cross-section [27].

For different applied pressures *P* ranging from 0.5 to 3 mTorr, the starting potential (V_B_) of the plasma for the OMSE reactor ranged from 300 to 240 V, while for the OMTSE reactor, it ranged from 400 to 220 V. This may be attributed to the large gap distance between the secondary anode and the cathode mesh (20 mm) for the OMTSE reactor, implying that the electron-neutral particle collision frequency ν_e-n_ was small and the mean free path λ_e-n_ was large. Therefore, the ionization probability in OMTSE was lower than that in OMSE.

Furthermore, the slope of the I–V characteristic for OMSE was higher than that for OMTSE, which means that the resistance and the resistivity of the discharge for the sample in OMSE decreased dramatically in comparison with OMTSE.

#### 3.1.2. Paschen Curves

The relationship between the product Pd as a function of the breakdown potential V_B_, i.e., V_B_ calculated as a function of Pd, is known as Paschen’s law [28], where d (cm) is the gap discharge between the electrodes, equal to 4 mm for OMSE and 15 mm for OMTSE, and P (mTorr) is the gas pressure.

**Figure 5:**
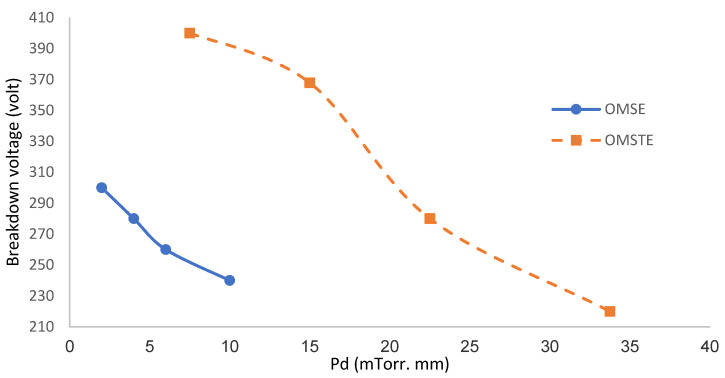
Paschen curve for OMSE and OMTSE, the breakdown voltage V_B_ as a function of Pd (mTorr. mm).

The Paschen curves in Figure 5 show that by increasing Pd (mTorr. mm) for both reactors, the breakdown voltage V_B_ began to decrease gradually (left-hand side of the Paschen curve). The V_B_ for OMSE was lower than for OMTSE, which may be attributed to the following:

(i) The small gap discharge for OMSE, where plasma was confined above the cathode mesh, leading to a decrease of the ionization coefficient and to a higher recombination coefficient of Ar_2_^+^ (0.7 × 10^−6^ cm^3^/s), whereby argon molecules suffered inelastic collisions with energetic electrons, excitation, and ionization when entering the discharge [29].
(ii) The collision frequency between electrons and neutral atoms or molecules in the gap discharge, which increased more for OMSE than for OMTSE [30].
(iii) The large gap discharge in the OMTSE reactor between the cathode mesh with respect to the secondary anode electrode, where the ionization cross-section decreased, and electrons needed more energy to reach the secondary anode [31].

#### 3.1.3. Current Density

Experimentally, the current density can be calculated using the I–V characteristics of the OMSE and OMTSE reactors, dividing current discharge I (mA) by cathode mesh area (cm^2^), and as derived theoretically in our previous work [32], as in Equation (2):

(2) $$\frac{J}{P^{2}}=\left\{\frac{4\;\left[1+\left(\omega /\alpha \right)\right]\varepsilon_{ο}{\left(\frac{e\lambda_{i}}{M}\right)}^{1/2}\;{V_{c}}^{3/2}}{{\left(P.d_{c}\right)}^{5/2}}\right\}$$

where *J* is the total current density, *M* is the mass of the ion, *e* is the electron charge, *ε*_0_ is the free space permittivity, and *λ_i_* is the mean free path of the ion. Furthermore, *V_c_* is the potential of the regions over the mesh equal to E *d*_c_, where *d*_c_ represents the cathode fall thickness of the most intense glow zone apart from the mesh and can be calculated theoretically using Equation (3):

(3) $$d_{c}=\frac{1}{\alpha }\ln[\frac{1+(\omega /\alpha )}{\left(\omega /\alpha \right)}]$$

$\frac{\omega }{\alpha }$ is the average number of secondary electrons produced per ionizing collision in the gas [33], and *α* is the first Townsend ionization coefficient and equal to *η*E, where *η* represents the ionization efficiency, as in Equation (4) [33] :

(4) η =α /E = αpEp=A P e−B pEE

**Figure 6:**
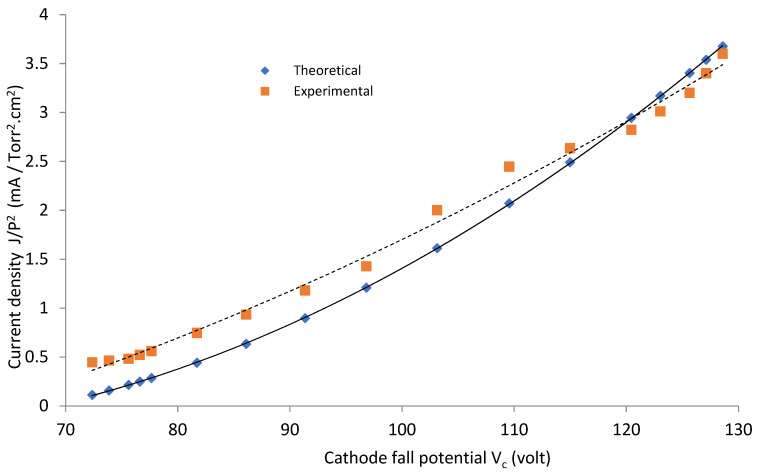
Current density as a function of the cathode fall potential for OMSE with applied pressure of 1 mTorr.

**Figure 7:**
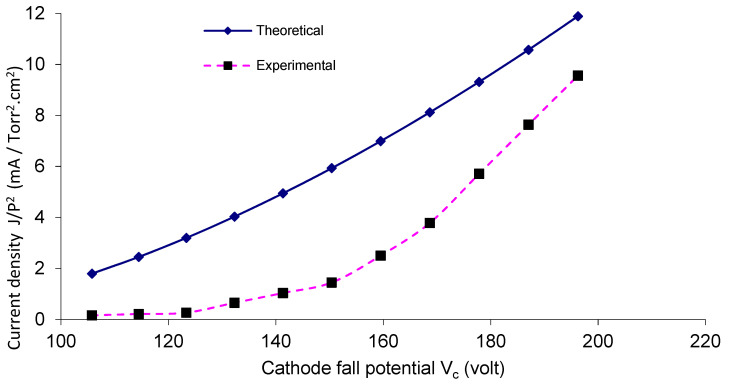
Current density as a function of the cathode fall potential for OMTSE with applied pressure of 1 mTorr.

For a gas pressure *P* equal to 1 mTorr, Figure 6 and Figure 7 show a comparison between the theoretical and the experimental results of current density J/p^2^ as a function of V_c_, for the two reactors OMSE and OMTSE, respectively, where J increased by increasing V_c_. The theoretical data are derived from Equation (2), where V_c_ is the potential of the regions over the mesh (apparently as the abnormal negative glow region in its characteristics).

The experimental value of OMSE ranged from 0.44 to 3.01 mA/cm^2^, in only slight agreement with the theoretical relations. The experimental value of OMTSE current density ranged from 0.15 to 9.5 mA/cm^2^, in partial agreement with the theoretical relations. This may be attributed to the increase in the confined sheath around the mesh wires for OMSE rather than OMTSE, where Ar molecules suffered inelastic collisions with energetic electrons. Moreover, more excitation and ionization processes took place, reducing the current density values for OMSE more than OMTSE [34].

Moreover, in the OMTSE low-pressure glow discharges, the experimental data and the theoretical curves of the current density agreed more than in OMSE. In the OMTSE case, this may be attributed to a dusty plasma produced from the contamination by polymerization [35] or by sputtering of the ions with the mesh.

#### 3.1.4. Cathode Fall Thickness

**Figure 8:**
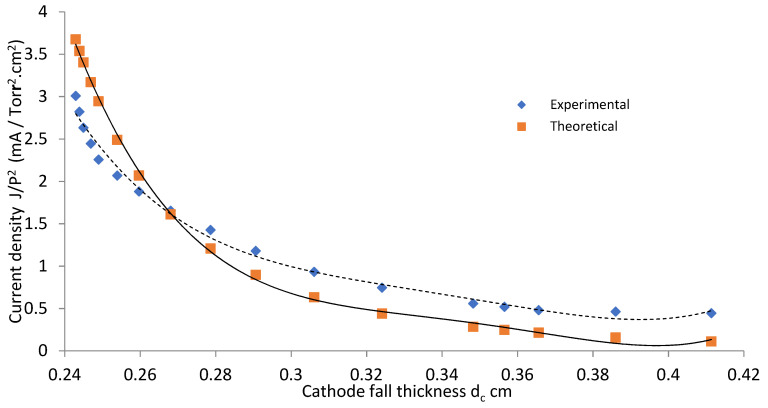
Current density as a function of the cathode fall thickness for OMSE with applied pressure of 1 mTorr.

**Figure 9:**
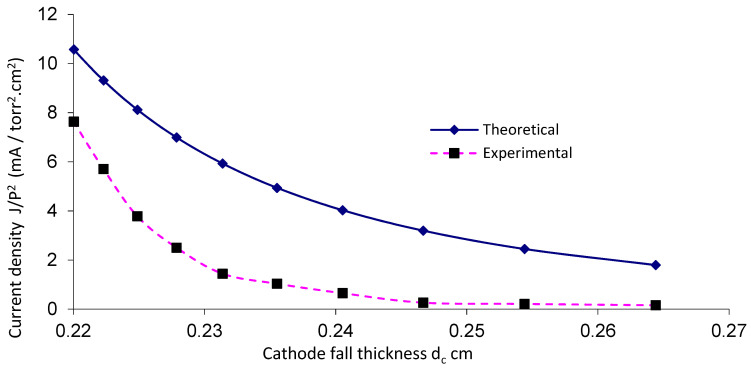
Current density as a function of the cathode fall thickness for OMTSE with applied pressure of 1 mTorr.

Figure 8 and Figure 9 show values of J/P^2^ as a function of the distance (d_c_) for OMSE and OMTSE, respectively, using Ar gas. The smallest value of cathode fall thickness (d_c_) corresponded to the largest value of the current density [36] (which referred to the closest regions where the samples were placed over the mesh). For OMSE, d_c_ was about 0.24–0.41 cm, while it was 0.22–0.27 cm for OMTSE. The experimental data agreed with the theoretical relations shown in Equation (3). For OMTSE, the experimental data partially agreed with the theoretical relations. The discrepancy between the experimental data and the theoretical curves at large values of V_c_, as shown in Figure 6 and Figure 7, may be attributed to the fact that a pure low-pressure argon discharge is a complex plasma at large values of V_c_, comprising electrons, ground state argon atoms, metastable argon atoms, argon ions, Ar_2_* and Ar_2_^+^ molecules (M* excitation process, M^+,−^ ionization process), and impurity atoms existing in argon or sputtered from electrodes [37].

### 3.2. The Influence of Different Cathode Configurations on the Surgical Gown

Under a gas pressure P equal to 1 mTorr using a DC glow discharge, surgical gown samples were exposed to uniform argon plasma and treated under the measured parameters of the two different configurations, OMSE and OMTSE, as follows:

(I) As seen in Section 3.1, the OMTSE current density ranged from 0.15 to 9.5 mA/cm^2^ for d_c_ ranging from 0.22 to 0.27 cm, and the OMSE current density ranged from 0.44 to 3.01 mA/cm^2^ for d_c_ ranging from 0.24 to 0.41 cm. The treatment efficiency was measured for the surgical gown surface in plasma over the mesh cathode at a distance equivalent to the cathode fall distance d_c_, and for a very short exposure time.
(II) From our previous work with the same construction mentioned in [23], the ion velocity ranged from 1 to 3.5 km/s for OMSE, and from 4 to 22 km/s for OMTSE, while the ion density N_i_ per unit area for OMSE was in the range of 10^9^ cm^−3^ and lower than that for OMTSE (in the range of 10^10^ cm^−3^).

#### 3.2.1. Performance Quality of the Surgical Gown

**Figure 10:**
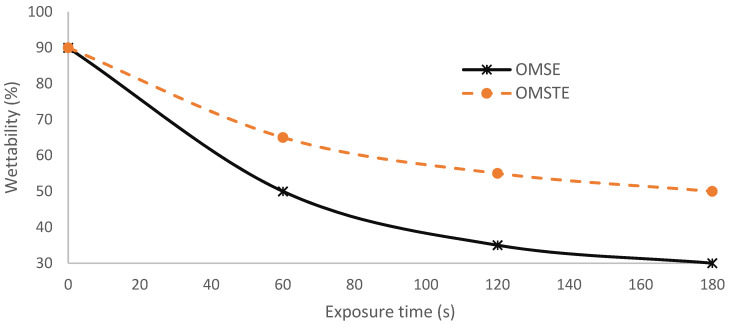
The wettability of the surgical gown as a function of exposure time for the different plasma cell configurations OMSE and OMTSE with applied pressure of 1 mTorr and cathode fall thickness of 0.25 cm.

Figure 10 shows the effect of the plasma cell configurations for OMSE and OMTSE on the wettability of surgical gown ω as a function of exposure time at applied low pressure, 1 mTorr [38]. The performance qualities of the surgical gowns for OMSE and OMTSE configurations were compared by measuring the wettability at different exposure times ranging from 0 to 180 s, where different surgical gown samples were placed over the mesh cathode on an axial moveable grounded holder 0.25 cm away from the mesh, the cathode fall thickness d_c_ range of both OMSE and OMTSE configurations. Moreover, the wettability ω at exposure time 180 s decreased from 90 to 50% for OMTSE and decreased from 90 to 10% for OMSE. This means that the wettability of the surgical gown decreased when increasing the treatment exposure time. This indicated the following:

(i) The treatment processes of the surgical gown exposed to plasma are described as follows [39,40]: Electrons and ions formed because of the plasma discharge. The sample was initially negatively charged, relative to the plasma bulk, because of the higher mobility of the lighter electrons. Then, more electrons were repelled from the sample and the positive ions were accelerated toward it.
(ii) The wettability of the modified surface decreased when decreasing the gas pressure, increasing the axial exposure distance (d_c_), and increasing the velocity of the penetrating species (ions, free electrons, neutral atoms, and molecules) on the textile surface [41]. This can be understood from Figure 8 and Figure 9 and Equation (2), where the cathode fall thickness increased with decreasing of the current density at low pressure, 1 mTorr.
(iii) The treatment efficiency reaches a maximum in plasma in a very short exposure time [42]; the poor wettability and maximum water repellency properties for OMSE, more so than OMTSE may be due to the apparent increase in the pressure and the change of the laminar mode for OMSE to turbulent mode for OMTSE because of the long distance between the mesh and the secondary electrode.

**Figure 11:**
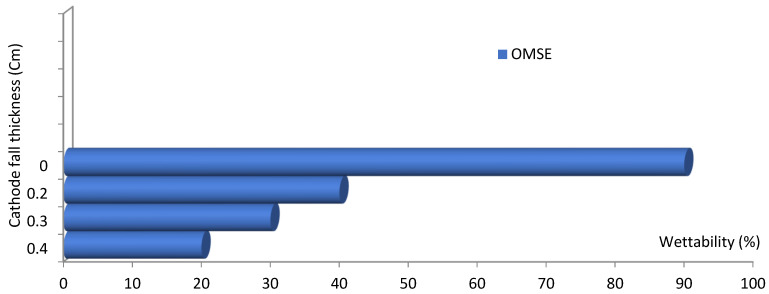
The wettability of the surgical gown (%) as a function of cathode fall thickness (cm) for plasma cell configuration OMSE at applied pressure of 1 mTorr and exposure time of 120 s.

Figure 11 shows the wettability of the surgical gown ω as a function of the axial distance from the mesh in the range of cathode fall thickness for the OMSE reactor at a constant argon pressure of 1 mTorr and short exposure time of 120 s, where the wettability decreased at the largest value of cathode fall thickness. This may be attributed to the following:

(i) More scattering of the positive ions and thus more chemical bonds broken by energy transfer from reactive particles to the sample surface, as a greater distance (d_c_) exposes a larger area of the sample [43,44,45].
(ii) The physical changes from the exposure to the plasma. These changes produce more reactive surfaces and affect wettability, as will be discussed in Section 3.3 [46].

#### 3.2.2. Mechanical Properties

**Figure 12:**
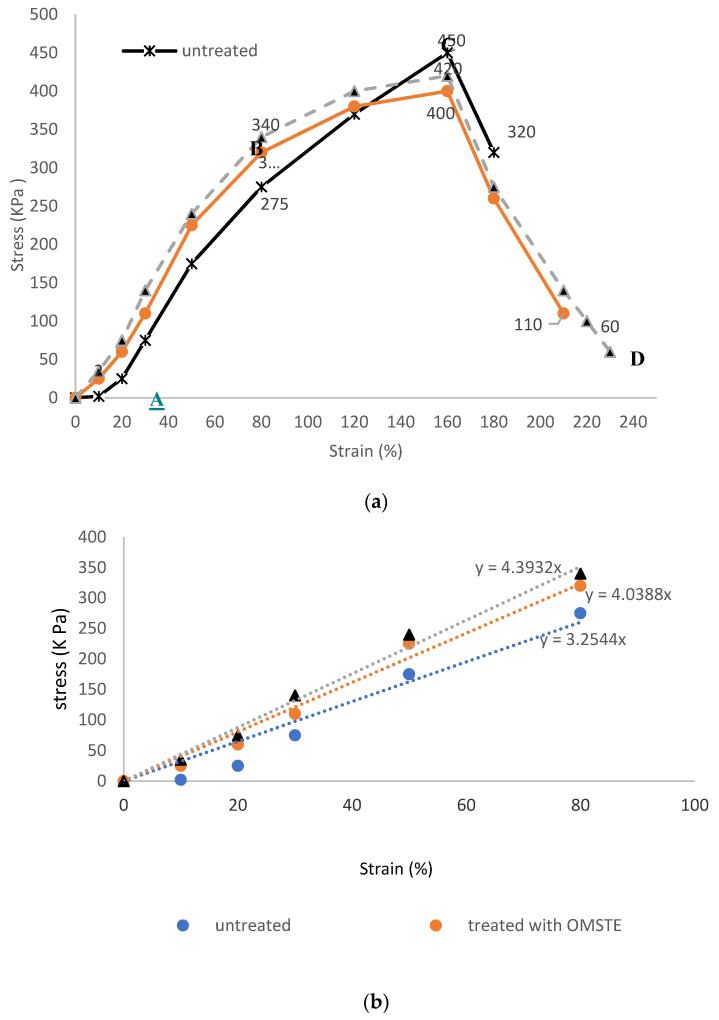
(**a**) The relation between stress  σ (KPa) as a function of the strain  ε (%) for untreated and treated samples with DC plasma reactors OMTSE and OMSE. (**b**) The elastic region AB, exhibiting straight lines, with the slope represents Young’s modulus (stiffness).

Figure 12a shows the tensile and the elongation behaviors for untreated and treated surgical gown samples for the two plasma reactors OMTSE and OMSE exposed to a uniform DC glow discharge of argon plasma to test their mechanical properties, as indicated by the stress  σ  (KPa) as a function of the strain  ε (%). Moreover, Figure 12b shows the linear region AB exhibiting straight lines represented by  σ=E ε, with the slope *E* representing Young’s modulus (stiffness) values. In the elastic region E  increased to 3.25 KPa for untreated samples, to 4.04 KPa for samples treated with OMTSE, and to 4.39 KPa for samples treated with OMSE [47].

Tensile resilience (RT) [48] is given by the area under the curve of the elastic region AB as in Equation (5):

(5) $$(\mathrm{RT})=\frac{1}{2}\sigma \;\varepsilon .$$

RT corresponds to values of 9000, 12,800, and 13,600 J/m^3^, for untreated, and treated with OMTSE and OMSE, respectively, indicating the better capacity of the surgical gown samples to absorb more energy when deformed elastically for OMSE samples than for OMTSE samples, as in Equation (6):

(RT)_OMSE_ > (RT)_OMTSE_ > (RT)_untreated_ (6)

WT, determined by the area under the stress–strain curve up to the fracture (breaking point) from A to D using Microsoft Excel, represents the energy required for extending the surgical gown length without damaging it and reflects the mobility of the garment under deformation (up to fracture) [49,50,51]. WT increased as follows: 51,695, 54,675, and 58,675 J/m^3^ for untreated and for treated with OMTSE and OMSE, respectively, as in Equation Equation (7):

(WT)_OMSE_ > (WT)_OMTSE_ > (WT)_untreated_ (7)

The mechanical properties of the untreated and treated samples are collected in Table 1, indicating the following:

(i) The mechanical properties of the surgical gown samples treated with plasma were more positively influenced in the OMSE reactor than in the OMTSE reactor.
(ii) The use of plasma to treat the surgical gown samples increased the elasticity area, the stretch, and the strain percentages.
(iii) The density and the energy of the positive ions emerging from the mesh and colliding with the surgical gown sample for OMSE were much greater than those for OMTSE. This can be attributed to the fact that there was a loss of energy for OMTSE due to (a) creation of a sheath around the mesh for OMTSE and (b) creation of dusty plasma due to more scattering in the longer distance between the mesh and the secondary electrode for OMTSE [52,53].

### 3.3. The Mechanisms of Plasma Interaction with Textile Surface

#### 3.3.1. Interaction Type

The interaction mechanism between the plasma species and textile materials mainly depends on modifications from the interaction between the plasma species and textile fibers, wettability, and mechanical properties of the surgical gown surface (with finished coded levels), which can be improved by the activation process [54]. The activation process helps to break the covalent bonds present on the surface of the surgical gown sample and generate radicals. These are highly reactive sites and combine with other species, such as organic molecules, unsaturated monomers, or reactive gases such as oxygen to generate functional groups on the surface [55]. Moreover, the activation process may be coupled with the etching process to clean the surgical gown surface with the ions bombarding the sample, which removes impurities and contaminants such as blood from the sample surface [56].

#### 3.3.2. Gas Type

When the excited argon species, viz., ions, electrons, meta-stables, and neutrals, bombard the textile surface along with energetic ultraviolet photons, they can break chemical bonds and initiate various reactions. Argon can change textile surface properties such as wettability and mechanical properties because of its high ablation efficiency and chemical inertness with the surface material [57]. Moreover, argon produces chain scissions on the surface (i.e., activation) and crosslinking through the reactions of inter- and intra-molecular polymer chains [58].

## 4. Conclusions

Two different configurations of the plasma cell cathode, namely, OMSE and OMTSE, were constructed and investigated theoretically and experimentally. In the OMSE reactor the optimum position of the sample with respect to the mesh was found to correspond to the cathode fall thickness (d_c_) and the smallest value of the current density suitable to modify the surface of the fabric sample. The area placed exactly over the mesh for OMSE was found to be the most intense glow zone. OMSE represented a suitable reactor for surface modification processes because of its steady and equilibrium plasma discharge.

At low pressure (1 mTorr) using a DC glow discharge, the wettability of the surgical gown decreased when increasing the treatment exposure time. The treatment resulted in poorer wettability and better water repellency properties for OMSE than for OMTSE because, as the cathode fall thickness increased, the current density decreased. For the OMSE reactor, the wettability of the surgical gown decreased at the largest value of cathode fall thickness at a farther axial distance from the mesh in the range of cathode fall thickness, where the resistance and the resistivity of the discharge for the sample decreased.

An experimental study of the performance quality and the influence of different cathode configurations of the plasma cell was performed regarding (a) the surface modification and performance quality of the surgical gown in low-density plasma using weakly ionized argon gas and (b) the analysis of the plasma reactive particles created in the glow discharge through the plasma–surface interaction process. The wettability of the surgical gown decreased when increasing the treatment exposure time. The treatment resulted in poorer wettability and better water repellency properties for OMSE than for OMTSE because, as the cathode fall thickness increased, the current density decreased. For the OMSE reactor, the wettability of the surgical gown decreased at the largest value of cathode fall thickness at a farther axial distance from the mesh in the range of cathode fall thickness, where the resistance and the resistivity of the discharge for the sample decreased.

All the mechanical properties of the untreated surgical gown samples and those treated with OMTSE and OMSE, such as the tensile strength and elongation percentage, ultimate tensile strength yield strength, strain hardening, resilience, toughness, and fracture, were measured.

Our future work will involve an experimental study of the plasma treatment, not only of direct physical effects and mechanical changes but also of the chemical changes caused by the plasma. The work will also involve conducting analytical investigations into the actual effect of the plasma treatment on the surgical gown.

## Figures and Tables

**Table 1:** The measured mechanical properties of the untreated samples and OMSE and OMTSE treated samples.

Units	Treated with OMTSE	Treated with OMSE	Untreated	Parameters	Position
KPa	4.04	4.39	3.25	stiffness	From A to B
KPa	320	340	225	Yield strength $\sigma_{Y}$	B
KPa	420	450	400	ultimate tensile strength $\sigma_{\mathrm{UTS}}$	C
KPa	100	110	175	strain hardening $\sigma_{\mathrm{UTS}}-\sigma_{Y}$	B–C
%	210	230	180	elongation percent at breaking point	D
J/m^3^	12,800	13,600	9000	resilience	Area under the curve of the elastic region
J/m^3^	54,675	58,675	51,695	toughness	Area under the stress–strain curve up to fracture

## Data Availability

Data are contained within the article.
